# Clinical Comparison of Full and Partial Double Pedicle Flaps with Connective Tissue Grafts for Treatment of Gingival Recession

**Published:** 2016-09

**Authors:** Ardeshir Ranjbari, Gholam Ali Gholami, Reza Amid, Mahdi Kadkhodazadeh, Navid Youssefi, Amir Reza Mehdizadeh, Maryam Aghaloo

**Affiliations:** 1 Periodontist, Private Practitioner, Ahvaz, Iran.; 2 Dept. of Periodontics, Dental School, Shahid Beheshti University of Medical Sciences, Tehran, Iran.; 3 Dental Research Center, Research Institute for Dental Sciences, Shahid Beheshti University of Medical Sciences, Tehran, Iran.; 4 Dentist, Dental Research Center, Research Institute for Dental Sciences, Shahid Beheshti University of Medical Sciences, Tehran, Iran.

**Keywords:** Root Coverage, Connective Tissue Graft, Pedicle Flap, Gingival Recession

## Abstract

**Statement of the Problem:**

Gingival recession has been considered as the most challenging issue in the field of periodontal plastic surgery.

**Purpose:**

The purpose of this study was to evaluate the clinical efficacy of root coverage procedures by using partial thickness double pedicle graft and compare it with full thickness double pedicle graft.

**Materials and Method:**

Eight patients, aged 15 to 58 years including 6 females and 2 males with 20 paired (mirror image) defects with class I and II gingival recession were randomly assigned into two groups. Clinical parameters such as recession depth, recession width, clinical attachment level, probing depth, and width of keratinized tissue were measured at the baseline and 6 months post-surgery. A mucosal double papillary flap was elevated and the respective root was thoroughly planed. The connective tissue graft was harvested from the palate, and then adapted over the root. The pedicle flap was secured over the connective tissue graft and sutured. The surgical technique was similar in the control group except for the prepared double pedicle graft which was full thickness.

**Results:**

The mean root coverage was 88.14% (2.83 mm) in the test group and 85.7% (2.75 mm) in the control group. No statistical differences were found in the mean reduction of vertical recession, width of recession, or probing depth between the test and control groups. In both procedures, the width of keratinized tissue increased after three months and the difference between the two groups was not statistically significant in this respect.

**Conclusion:**

Connective tissue with partial and full thickness double pedicle grafts can be successfully used for treatment of marginal gingival recession.

## Introduction

Gingival recession is among the most common periodontal problems in young adults.[1-2] Epidemiologic studies have shown that more than 50 percent of the population have one or more gingival recession sites of 1 mm or more.[3-4] Gingival recession can occur in patients with fair or poor oral hygiene. There is a clear relationship between gingival recession and several risk factors such as dental plaque, calculus, tobacco consumption, tooth brushing frequency, traumatic tooth brushing, high frenal attachment, trauma, and malposition of teeth.[5]

Complications of gingival recession include tooth sensitivity, esthetic problems, food impaction, and plaque accumulation leading to root caries, lack of attached gingiva, hyperemic pulp, endodontic problems, difficulties in restoration, and finally tooth loss.[6]

Currently, numerous researchers have attempted to treat marginal tissue recession. Treatments for gingival recession include gingival grafting,[7] guided tissue regeneration (GTR),[6] and orthodontic therapy.[8] Using gingival grafts for root coverage has a historical background. However, most studies on this subject were conducted in the second half of the 20th century.[7]

Different surgical techniques have been proposed and employed by researchers for root coverage such as laterally sliding flap,[9] free gingival graft,[10] sub-epithelial connective tissue graft,[11-12] coronally-positioned flap[13-15] and GTR.[16-17]

Studies showed that the mean root coverage (MRC) was not equal in different techniques. The MRC was reported to be 55-91.2% for coronally advanced flap (CAF), 43-85.3% for free gingival graft and 53.5-87.1% for GTR.[18]

The two latest methods applied successfully are subpedicle connective tissue graft and connective tissue with partial thickness double pedicle graft introduced by Nelson and Harris who are the pioneers of these methods, respectively.[19-20] They reported 91% and 97.7% MRT, respectively. The success of Harris’s technique was more than that of other procedures described earlier, which seems to be due to the mucosal flap design.[20-22]

Recent studies have shown that using connective tissue graft (CTG) in conjunction with CAF, modified coronally advanced flap (MCAF) or double pedicle graft (DPG) results in more complete root coverage (CRC) or MRC than the bio-absorbable membranes.[23] Thus, using CTG in different procedures is still recommended as the most efficient method for covering the denuded root surfaces.

One of the advantages of free connective tissue grafts is the healing by primary intention in the donor site. This is opposite to the healing process of free gingival grafts in which the donor site will be left without coverage, causing pain and discomfort for the patient during the healing process.[23]

This randomized double-blind controlled clinical trial aimed to comparing the clinical outcomes of two techniques in order to recommend a simpler and more efficient method. 

## Materials and Method

Eight patients (6 women and 2 men) aged 15- 58 years old (Mean: 34.2±14.2) with mirror image buccal Miller class I and II gingival recessions were included. According to the inclusion criteria, each and every patient had both types of defects in the oral cavity. The defects were randomly assigned into two groups with the toss of a coin.

The patients signed an informed consent. Those with systemic disease, smoking and drug usage causing gingival enlargement were excluded from the study. Twenty cases with Miller class I and II mirror image recession defects were randomly assigned into the test [partial thickness double pedicle graft (PTDPG)] and control groups [full thickness double pedicle graft (FTDPG)]. Two patients had four defects. Based on normality assumptions for each group, assuming that they have the same common variance, the calculated power analysis for this study was 0.784.[24]

**Pre-surgical procedures**

Phase 1 therapy included oral hygiene instruction, besides scaling and root planning with hand instrument. In case of any occlusal interference, occlusal adjustment was done. Two weeks after phase 1 therapy, the patients were asked to return for oral hygiene monitoring. For this purpose, simplified debris index was used as a measure. If the score was 2 or 3 (soft debris covered more than 1/3 of the denuded root), the surgery would not be performed.

The pre-surgical procedures consisted of preparation of surgical acrylic stent, taking parallel radiographs, intraoral photography from recession sites before, during and after the surgery, and measurements after the surgery. The examiner was a periodontist with 15 years of professional experience. The clinical parameters including recession height, recession width, pocket depth, and keratinized tissue width (KTW) were measured before and after the surgery. Both the patient and examiner were blind to the method of treatment for each mirror defect site.

**Surgical procedures**

**PTDPG with connective tissue (test group)**

**Figure 1:**
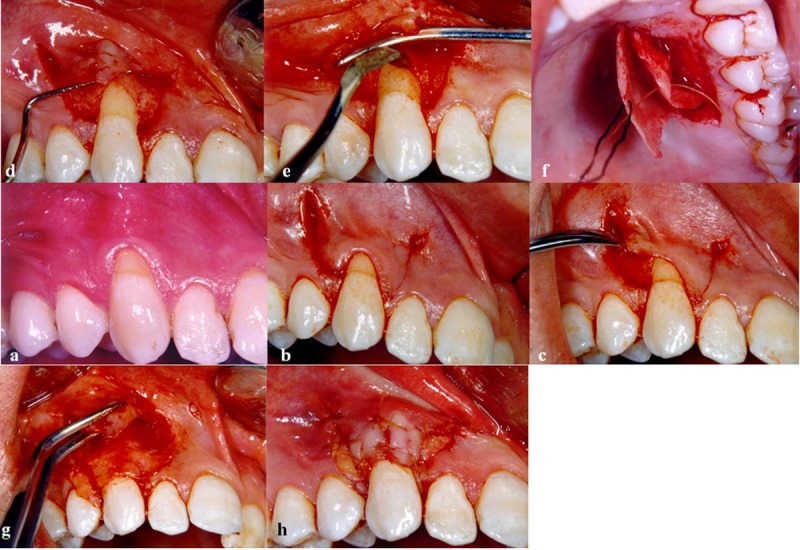
a: Tooth #6 with 3 mm class I Miller recession , treated by full thickness double papillary graft (FTDPG) technique considered as control group,  b: Vertical incisions at the level of cementoenamel junction without involving the neighboring teeth,  c: Raising the FTDPG by using periosteal elevator, d: Suturing the double pedicle in order to make a single pedicle graft,  e: Flattening the root surface by using surgical hoe,  f: Harvesting connective tissue from palatal region, g: Suturing the connective tissue over the denuded root, h: Covering the connective tissue with double papillary flap

All patients were instructed to use 2 tablets of Ibuprofen 400 mg one hour before surgery and one tablet every 8-12 hours in case of having pain after the surgery. After injection of the anesthetic drug, the denuded root was planned. The purpose of this action was to remove any possible calculus, caries, or root concavity (Figure 1a).

Any frenal pull was removed by a surgical blade. A horizontal incision was made at the level of cementoenamel junction at about 0.5-mm distance from the mesial and distal margins of the defect in order to avoid further recession.

Vertical incisions were made starting from the two ends of these horizontal incisions and were extended to the alveolar mucosa (Figure 1b). A sulcular incision connected these two horizontal incisions and the pedicle flaps were reflected with a surgical blade up to a position which allowed its mesial or distal movement. If functional movements were affecting flap displacement, the flap was further elevated by the use of 5-0 resorbable suture. The pedicle flaps were sutured to make a single flap. The connective tissue was harvested from the palate by trap-door technique. In the palate, the distance between the horizontal incision and gingival margin had to be more than 2 mm. The epithelium was elevated by using no.15 scalpel and then a 1.5-2.0-mm thick connective tissue graft was obtained. The epithelium was laid back and sutured with 3-0 silk suture. The connective tissue was trimmed and placed over the recession area up to the cementoenamel junction level and secured in place by 5-0 absorbable suture. Then the pedicle flap covered the connective tissue up to 2/3 of it by 5-0 absorbable sling suture, and wet gauze was kept in place for 10-15 seconds. The surgical area was covered by a dressing.

**FTDPG with connective tissue (control group)**

The procedure was the same as that of the test group except for the flap which was mucoperiosteal and was reflected by using a periosteal elevator (Figure 1c-h).

**Post-surgical care**

All patients were instructed to rinse their mouth with 0.2% chlorhexidine gluconate mouthwash for 60 seconds twice daily for 4 weeks. Amoxicillin (500 mg capsules) was prescribed every 8 hours for one week. The palatal sutures were removed after one week. Recall appointments for professional supragingival tooth cleaning were scheduled at 1, 2, 4, 8, and 12 weeks. For the first 4 weeks post-surgery, the patients were instructed to clean their teeth crowns with a cotton swab and chlorhexidine mouthwash 0.12%. They were also instructed to carefully perform flossing the teeth in the surgery site four weeks after the surgery. After 12 weeks, the recession height, recession width, sulcus depth, width of keratinized tissue, and clinical attachment level were measured by the same operator. Intraoral photographs were also obtained. Student’s t-test was used to compare the two groups in terms of the mean values of parameters at the baseline. The specific indication for use of student’s t-test was the equality of variances of parameters. Thus, the hypothesis for equality of variances in the 2 groups was tested by using the Levene’s test. Paired t-test was used for intragroup comparison of the mean values before and 6 months after the surgery. 

## Results

**Figure 2:**
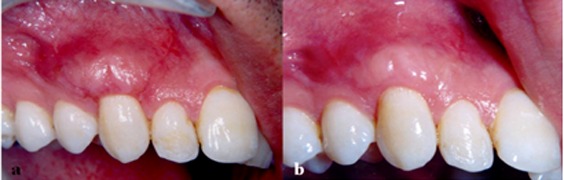
: The clinical view manifesting surgical region three weeks after healing, b: The clinical view manifesting surgical region four months after healing

**Figure 3:**
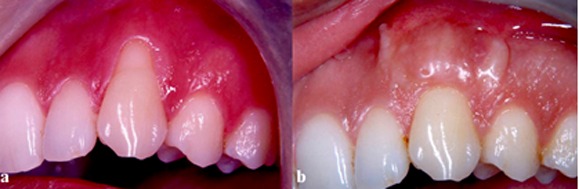
: Tooth #11 before treatment with partial thickness double Pedicle graft (test group) at the base line,  b: Tooth #11 four months after healing

Data analysis showed no statistically significant difference between the two groups in this respect. The pre-operative parameters were not statistically significantly different in the two groups as revealed by Levene's Equality of Variances test. Hence, the groups were proven to be comparable (Table 1). As demonstrated in tables 2 and 3, statistically significant differences were detected in the mean and standard deviation of parameters in both groups except for pocket depths. The MRC was 2.75 ± 0.87 mm (85.7%) and 2.83 ± 0.68 mm (89.14%) in the control and test groups, respectively. There was no statistically significant difference between the test and control groups in terms of root coverage. In the test group, 7 out of 10 defects (70%) showed CRC; whereas, it was 6 out of 10 defects (60%) in the control group. Fisher’s exact test showed no statistically significant difference between the two groups in terms of CRC (figures 2 and 3).

**Table 1:** Mean value of parameters before surgery in the control and test groups (FTDPG)

**Parameters** ** Groups**	**(W.K.G) (mm)**	**(P.D) (mm)**	**(C.A.L) (mm)**	**(H.R) (mm)**	**(V.R) (mm)**
PTDPG (test)	2.72 ± 1.78	1.35 ± 0.78	4.49 ± 0.79	4.1 ± 0.52	3.14 ± 0.39
FTDPG (control)	2.65 ± 1.18	1.26 ± 0.36	4.45 ± 0.55	4.07 ± 0.52	3.19 ± 0.44
T-Test	NS	NS	NS	NS	NS

**Table 2:** Mean value of parameters before and 3 months after surgery in the test group (PTDPG)

**Parameters** ** Groups**	**(W.K.G) (mm)**	**(P.D) (mm)**	**(C.A.L) (mm)**	**(H.R) (mm)**	**(V.R) (mm)**
Before surgery	2.65 ± 1.18	1.26 ± 0.36	4.45 ± 0.55	4.07 ± 0.55	3.19 ± 0.44
Three months after surgery	4.15 ± 1.18	0.9 ± 0.52	1.16 ± 0.61	0.88 ± 1.42	0.36 ± 0.63
Paired t-test	*p*< 0.01	NS	*p*< 0.0005	*p*< 0.0005	P<0.0005

**Table 3:** Mean value of parameters before and 3 months after surgery in the control group (FTDPG)

**Parameters** ** Groups**	**(W.K.G) (mm)**	**(P.D) (mm)**	**(C.A.L) (mm)**	**(H.R) (mm)**	**(V.R) (mm)**
Before surgery	2.72 ± 1.79	1.35 ± 0.78	4.49 ± 0.79	4.1 ± 0.52	3.14 ± 0.39
Three months after surgery	4.15 ± 1.18	1.00 ± 0.53	1.39 ± 0.57	1.32 ± 1.74	0.39 ± 0.52
Paired t-test	*p*< 0.02	NS	*p*< 0.0005	*p*< 0.0005	*p*< 0.0005

The keratinized tissue width seemed to be sufficient in all cases. Keloid-like appearance was not detected in any case. In one case, the surgeon decided to proceed with gingivoplasty which has the advantage of no color difference between the grafted area and the neighboring tissue. The final appearance of gingiva was acceptable and the color of the grafted area was compatible with that of the neighboring tissues. The resulting appearances were almost acceptable after the surgery. However, if a patient had desired a more esthetic outcome, laser-assisted procedures were considered as a supplementary therapy. 

## Discussion

The purpose of this study was the clinical comparison of full thickness double pedicle flap and partial thickness double pedicle flap along with the connective tissue graft for treatment of gingival recession. The results showed that both techniques were effective and predictable for treatment of marginal tissue recession. The MRC was 88.14% in PTDPG and 85.7% in FTDPG; and the CRC was 70% and 60% in the two groups, respectively.

Different techniques have been introduced for root coverage with acceptable results. Since our hypothesis was based on superiority testing, the study results failed to show significant differences, as CRC was 70% and 60% in the two groups, respectively. Moreover, despite the 4% difference between the two groups, we could not test the equivalence hypothesis for the two techniques due to the limited sample size. Further investigations in this regard need to be done on a larger sample size.

Mazzocco *et al.*[25] in a similar study demonstrated that using sub-epithelial connective tissue graft (SCTG) was effective in gingival recession. The MRC in 6 months was 96%. They also investigated the difference between FTDPG and PTDPG. The MRC was 97% in FTDPG and 95% in PTDPG. The results showed no statistically significant difference between the two groups in pocket depth reduction or KTW increase. Their findings confirmed those obtained by the study of Harris in 1994. Harris[20] examined the outcome of connective tissue graft along with PTDPG on 100 treated defects. He reported a MRC of 97.7%. He also demonstrated that creeping attachment occurred in 95.5% of defects and CRC was obtained in 77.3% of them.[22]

Using SCTG with different surgical techniques indicated that it can be considered as a gold standard procedure.[23, 25] In a systematic review, Hofmänner *et al.*[23] revealed the effectiveness of using CTG in combination with different techniques in improving CRC and MRC. Their study indicated that MCAF+ CTG might improve the long-term stability of CRC compared with MCAF. The CRC was 52% in MCAF+CTG, but without CTG, this rate reduced to 35%.[23] In another systematic review, Chambrone *et al.*[26] detected a statistically significant reduction in gingival recession with SCTG when compared to acellular dermal matrix grafts and GTR with restorable membrane. Furthermore, a statistically significant increase was observed in KTW with SCTG compared to GTR with restorable membrane.

Mahajan *et al.*[27] compared the periosteal pedicle graft with subepithelial connective tissue graft. They reported that the defect coverage in periosteal pedicle graft was 3.1+0.13 mm (92.6%). This rate was 2.7+0.11 mm (88.5%) in SCTG group. Differences between the two groups were significant, showing more patient satisfaction and comfort during and after the procedure in the periosteal pedicle group. In a long-term study, Lee *et al.* evaluated the parameters like gingival recession clinical attachment loss, width of keratinized gingiva and MRC for subpedicle free connective tissue graft. The GR decreased from 3.67+0.58 mm at the baseline to 0.33+0.43 mm at 36 months; the MRC was 91.28% in this time period. The clinical attachment loss and width of keratinized gingiva also changed significantly. The most positive outcomes at 12 months were observed in gingival recession, clinical attachment loss, and width of keratinized gingiva; they were maintained at stable levels within 36 months of the study.[12]

Many recent studies investigated the efficacy of guided tissue regeneration in comparison with other techniques for treatment of gingival recession. Ruccuzzo *et al.*[28] showed significant recession reduction in CTG compared to GTR and differences between CAF and GTR were not significant. In a 5-year observation, Dominiak *et al.*[29] compared the efficiency of three surgical methods including double pedicle bilateral flap, coronally repositioned flap in combination with CTG (CRF_CTG) and coronally advanced flap in combination with GTR using collagen membrane (GTR_CM). Average percentage of root coverage with GTR_CM was 90% followed by 82% with CRF_CTG. Rosetti *et al.*[30] compared SCTG and GTR and found that both procedures were statistically similar in terms of root coverage (SCTG=95.6%, GTR=84.2%). The SCTG was significantly better than GTR in terms of GR and keratinized tissue. Although we used subpedicle connective tissue graft in the present study, our results are comparable with those of subepithelial CTG. The rationale of using bilayer techniques is to improve the blood supply.

Our results showed 2.75±0.87 mm mean root coverage after 6 months in FTDPG (85.7%), and 2.83±0.68 mm in PTDPG (89.14%) group. These rates are comparable with the findings of previous investigations.[19, 21] Nelson[19] reported 91% MRC, which is higher than our result (85.7%) obtained by the same technique (FTDPG). This difference may be due to the study duration (6 months in our study and 42 months in Nelson’s study); during this period, creeping attachment might have occurred. This phenomenon is defined as the coronal migration of marginal gingiva after surgery, where the root is denuded.[30] The same phenomenon has been reported and confirmed by other studies.[22] For this reason, by increasing the duration of follow-up visits, different results may be obtained. According to Matter, this happens one month after the surgery and may continue for more than a year.[31-32] The amount of horizontal recession before the surgery is one of the factors influencing the outcome of treatment. In some studies, horizontal recession was not considered as a criterion.[19] In our study, the mean preoperative horizontal recession in FTDPG group was 4.1±0.52 mm which was equal to the wide group according to the classification of Sullivan and Atkins.[33]

In the present study, PTDPG showed mean root coverage of 89.14%; whereas, this rate was 97.4% in Harris’s study.[20] This difference may be due to the sensitivity of techniques and/or usage of tetracycline hydrochloride as root conditioner in the other study. The current study did not use tetracycline hydrochloride in order to reduce the confounding factors. According to some researchers, using tetracycline as root conditioner during surgery can reinforce connective tissue attachment to the denuded root surface, although supporting data is scarce.[34] Thus, additional studies on the effect of tetracycline as root conditioner seem necessary. Harris[21] also reported creeping attachment even after one year post-surgery; therefore, further studies are required to determine the occurrence of creeping attachment in long-term. To sum it up, although some clinicians claimed that chemical root surface conditioning positively influenced flap adhesion, its effectiveness was unpredictable for various surgical methods performed in studies with larger sample size.[35-36]

This study failed to show a significant difference between PTDPG and FTDPG in clinical parameters like KTW, pocket depth, and clinical attachment level which is in agreement with the results of Mazzocoo *et al.*[25] In Both PTDPG and FTDPG, the gingival tissue was firmly attached to the root surfaces, as probing pocket depth after 6 months was 1.00±0.53 mm in FTDPG, and 0.9±0.52 mm in PTDPG. It means that sulcus depth before and 6 months after the surgery was not significantly different between the two groups. There are some basic differences between these methods, although the similarity in clinical parameters is apparent. It is not technically easy to achieve a fine partial-thickness flap particularly in a thin periodontal biotype. Progressing towards an excessively thin flap increases the risk of serious complications such as perforation and even consequent necrosis of surrounding tissue. The blood supply from the inner part of the flap together with that of the bone is sufficient for survival of the graft and for achieving an outcome comparable to that achieved with a partial-thickness flap reflection. Although the most success rates in root coverage therapy owes to the partial thickness method, this method is immensely technique-sensitive and requires great expertise and skill. On the other hand, performing this type of flap surgery would not be favorable in many cases due to the thinness of available soft tissue and possibility of sudden lacerations. Therefore, clinical comparison of full and partial thickness methods is noble and essential.[20, 22]

Despite the similarities in clinical parameters and some differences in basis of the techniques, more studies are recommended to evaluate the differences in blood supply of grafts in these methods. 

## Conclusion

Both FTDPG and PTDPG techniques are effective for treatment of gingival recession and can significantly increase the gingival level. There are no significant differences between these two methods but some factors can influence the result such as using tetracycline hydrochloride as a root conditioner and also the long-term  follow-up i.e. more than 6 months. More studies are recommended to evaluate the differences in blood supply and histologically analyze the type of attachment in the treated area. 
